# Targeted Drug Administration to Reduce Malaria Transmission: A Systematic Review and Meta-Analysis

**DOI:** 10.4269/ajtmh.22-0754

**Published:** 2024-01-23

**Authors:** Maria Tusell, Elisabet Martí Coma-Cros, Beena Bhamani, Vita Mithi, Elisa Serra-Casas, Nana Aba Williams, Kim A. Lindblade, Koya C. Allen

**Affiliations:** ^1^ Barcelona Institute for Global Health (ISGlobal), Hospital Clínic – Universitat de Barcelona, Barcelona, Spain;; ^2^ Armref Data for Action in Public Health Research Consultancy, Mzuzu, Malawi;; ^3^ Society for Research on Nicotine and Tobacco – Genetics and Omics Network, Madison, Wisconsin;; ^4^ Leaders of Africa Institute, Baltimore, Maryland;; ^5^ Global Malaria Programme, World Health Organization, Geneva, Switzerland

## Abstract

In low– to very low–malaria transmission areas, most infections may be accrued within specific groups whose behaviors or occupations put them at increased risk of infection. If these infections comprise a large proportion of the reservoir of infection, targeting interventions to these groups could reduce transmission at the population level. We conducted a systematic review to assess the impact of providing antimalarials to groups of individuals at increased risk of malaria whose infections were considered to comprise a large proportion of the local reservoir of infections (targeted drug administration [TDA]). A literature search was conducted in March 2021 and updated in April 2022. Two reviewers screened titles, abstracts, and full-text records. The Grading of Recommendations Assessment, Development and Evaluation approach was used to rate the certainty of the evidence (CoE) for each outcome. Out of 2,563 records, we identified five studies for inclusion: two cluster-randomized controlled trials (cRCTs) in Uganda and Kenya; one controlled before-after study in Ghana; and two uncontrolled before-after studies in Sri Lanka and Greece. Compared with no intervention, TDA resulted in little to no difference in the prevalence of infection at the population level (risk ratio [RR]: 0.85, 95% CI: 0.73–1.00; one cRCT, high CoE), although TDA likely resulted in a large reduction in prevalence among those targeted by the intervention (RR: 0.15, 95% CI: 0.06–0.38; two cRCTs, moderate CoE). Although TDA may reduce the burden of malaria among those receiving antimalarials, we found no evidence that it reduces transmission at the population level.

## INTRODUCTION

As malaria transmission decreases, risk of infection becomes more heterogeneous and malaria cases tend to concentrate in certain locations or groups of individuals. This results in a specific proportion of the population accounting for the majority of the malaria burden in an area and for sustaining transmission.^1^^,^^2^ Countries approaching malaria elimination often find most of their remaining cases clustering in specific geographic areas that are highly receptive to malaria or concentrated within specific groups of individuals whose activities, behaviors, or occupation put them at increased risk of exposure to malaria vectors. Risk factors can include age, sex, or social characteristics; occupations such as mining, farming, military, or laborers; or behaviors such as participation in outdoor leisure activities, traveling, and migrant populations.^3^^–^^5^ In this context, it becomes increasingly important to identify these groups at increased risk of infection and to implement interventions directed toward them (i.e., targeted strategies).^6^ With targeted strategies, we could capture a significant proportion of the human malaria parasite reservoir in a specific area and therefore reduce malaria transmission. This reduction in transmission would then benefit not only those who receive the intervention but also the wider community. Therefore, in situations where high-risk groups can be easily identified and are known to harbor a significant proportion of the human malaria parasite reservoir,^7^^,^^8^ administering chemoprevention to only these individuals, rather than to the entire population as is done in mass drug administration, may be a more cost-effective and acceptable approach to reduce the reservoir of malaria infection and decrease transmission. This review focuses on targeted drug administration (TDA), also known as targeted treatment, a form of chemoprevention that involves the administration of a full therapeutic course of an antimalarial medicine to a subpopulation at higher risk of infection irrespective of infectious status.^5^

To some extent, TDA is similar to other intermittent preventive treatment (IPT) interventions that target pregnant women or children, as they are the populations at high risk of infection. However, the distinction between IPT and TDA is that the former, IPT, is meant to reduce the consequences of infection in high-risk groups (through prevention and treatment), whereas the latter, TDA, is meant to reduce population-level transmission by reducing the reservoir of infection. One of the main principles behind TDA is the ability to identify and administer medicines to specific groups at increased risk of infection who may constitute a large proportion of the infections in an area. It is expected that the aforementioned scenario would be more common when transmission declines to a low or very low level. Therefore, if proven effective, TDA could be an intervention to reduce both the malaria burden of the individuals targeted by the intervention as well as transmission at the population level.

In support of the WHO guideline development process, we conducted a systematic review of the effectiveness of TDA to reduce malaria transmission. In particular, we aimed to determine the benefits and harms of administering a therapeutic course of antimalarial medicine to adults and children at higher risk of malaria infection in areas with ongoing transmission or malariogenic potential compared with no intervention. In addition, we summarized available evidence on relevant contextual factors, which are additional aspects beyond benefits and harms that address circumstances that can affect the implementation and impact of an intervention and that are considered by the WHO when recommendations to provide a health systems perspective are developed. We aimed to also summarize insights from mathematical modeling studies, if available.

## MATERIALS AND METHODS

This systematic review followed the Preferred Reporting Items for Systematic Reviews and Meta-Analyses (PRISMA) format,^9^ and the protocol was registered in the International Prospective Register of Systematic Reviews (CRD42021237822).^10^

Complete details of the eligibility criteria, study selection, data collection, and analysis have been described extensively elsewhere.^11^ An overview of the methods is provided below.

### Population, intervention, comparison, and outcomes.

This review aimed to answer the following question: “What are the relative effects (benefits and harms) of TDA compared with no TDA in adults and children at increased risk of malaria infection?” The inclusion and exclusion criteria were set by a population, intervention, comparison, and outcomes (PICO) model for evidence-based research. The targeted populations included adults and children who were defined as being at high risk of infection based on demographic, occupational, or other exposure characteristics, including recent travel to a higher transmission area. Special attention was paid to studies that targeted populations thought to be important reservoirs of infection, such as migrant laborers or refugees originating from a malaria endemic region, or populations known to be the remaining foci of transmission in areas approaching elimination, such as forest workers in the Greater Mekong subregion.^4^ Studies targeting schoolchildren were also included as school-aged children were considered a high-risk group and it has been shown that they are significant reservoirs of infection.^12^^,^^13^ For the purpose of this review, TDA was defined as the administration of a full therapeutic course of an antimalarial medicine to a subpopulation at high risk of infection irrespective of infection status,^5^ including treatment of *Plasmodium vivax* and *Plasmodium ovale* liver-stage parasites, to reduce malaria transmission at the population level. All transmission intensity settings were included. Targeted drug administration was compared with no TDA, and the outcomes of interest, described below, were measured at the population level and among individuals receiving the intervention. For randomized studies, only cluster-randomized trials were included under the assumption that to capture the potential impact of TDA on transmission, key outcomes should be measured at the community level rather than the individual level. Strategies that were applied to a large geographic area (i.e., mass strategies) and strategies that respond to individual cases (i.e., reactive strategies) were not included in this review.

### Selection of studies.

Inclusion and exclusion criteria were developed based on the PICO model to determine the selection of studies for review. Studies had to report at least one of the following to be included: outcomes measured at the population level including incidence and prevalence of infection, elimination (defined as zero indigenous or local cases for a period during the transmission season), incidence of clinical malaria, and drug resistance and outcomes measured among the group receiving the intervention, including adverse events (AEs) and prevalence of infection. Prevalence of infection among the group targeted by the intervention was included to serve as a proxy indicator of the potential effect at the population level.

Any study that met the intervention and population inclusion criteria as defined in the PICO question that also reported on contextual factors (namely values and preferences, acceptability, health equity, financial and economic considerations [resource use], and feasibility)^14^ was selected, and information was then summarized for those contextual factors that were addressed.

### Data extraction, analysis, and assessment of quality.

The search strategy for this review is presented in Supplemental Table 1 and was conducted in March 2021 and updated in April 2022. After removing duplicates, two review authors independently reviewed each study for eligibility. The data collection process, assessments of risk of bias in individual studies, data synthesis, assessment of heterogeneity, and assessments of quality were conducted following the methods described elsewhere.^11^ In short, study review for inclusion and data extraction was conducted using EPPI-Reviewer, a systematic review software, and a validated Excel data extraction tool. The risk of bias was assessed using the Cochrane Risk-of-Bias Tool for randomized trials^15^ and the Risk of Bias Tool in Nonrandomized Studies – of Interventions (ROBINS-I), respective to the study designs of included studies.^16^ We assessed the effect of the intervention using odds ratios, risk ratios (RRs), or rate ratios. To combine data, we used fixed-effects meta-analysis. The Grading of Recommendations, Assessment, Development and Evaluations (GRADE) approach was used to rate the certainty of the evidence (CoE).^17^^,^^18^

## RESULTS

**Figure 1.**
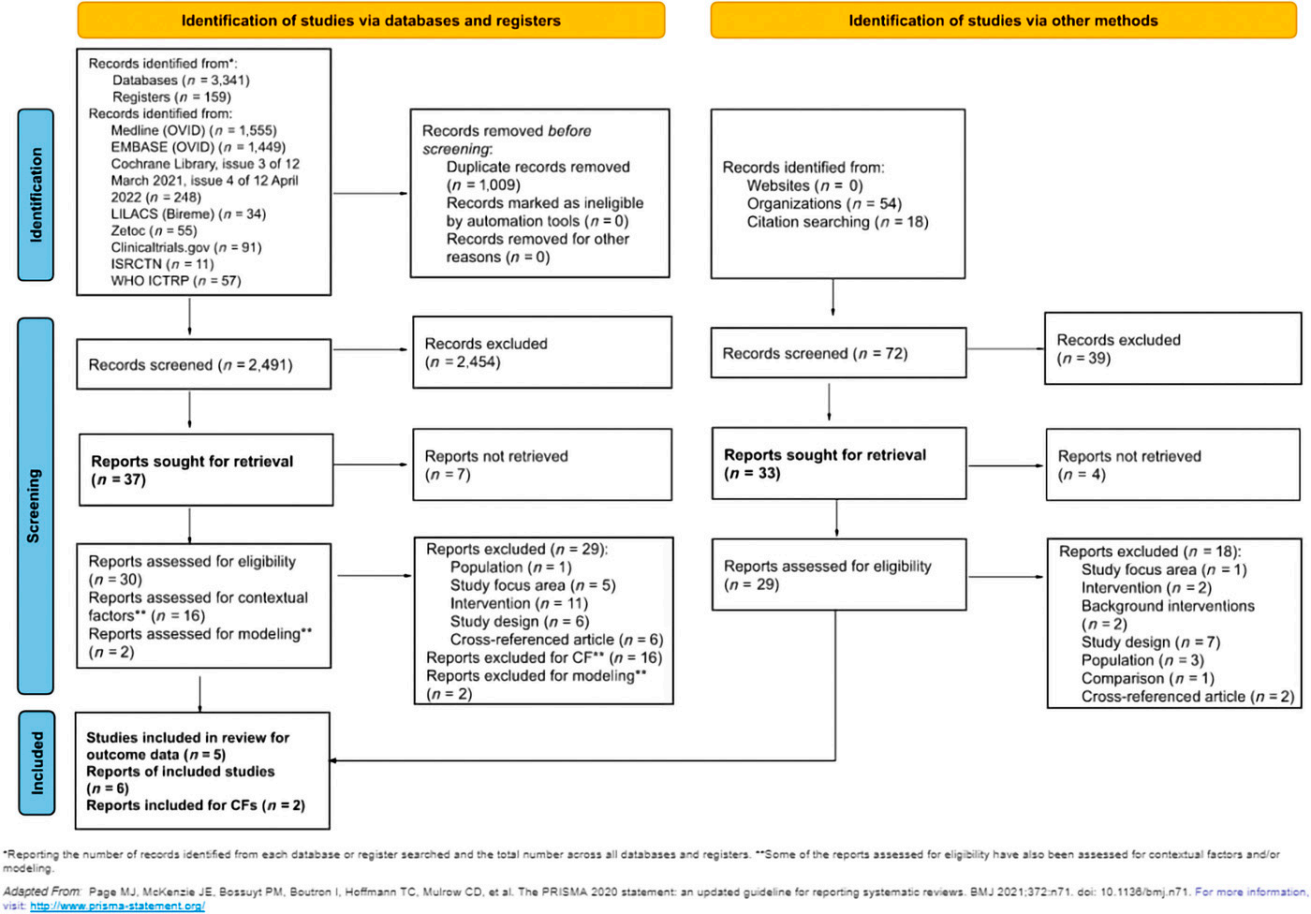
Preferred Reporting Items for Systematic Reviews and Meta-Analyses (PRISMA) diagram. Bold text indicates total number of records assessed at the full-text stage (screening phase) and total number of records included in the review (included). CF = contextual factors; ICTRP = International Clinical Trials Registry Platform; ISRCTN = International Standard Randomised Controlled Trial Number.

A total of 3,572 records were identified: 3,341 via searching databases, 159 from registers, and 72 via other methods (e.g., websites, organizations, and citation searching). Before the screening, 1,009 records were removed because of duplication, with a total of 2,563 records to be screened against title and abstract eligibility (2,491 identified via databases and registers and 72 identified via other methods). Of these, 70 were assessed for full-text eligibility criteria (37 from the database search and 33 identified via other methods). After the full-text screening, five studies were included for assessment of outcome data (six reports) and two studies were included for assessment of contextual factors. The detailed PRISMA flow diagram is presented in Figure 1.

There were 62 articles excluded after full-text screening owing to the following reasons: did not meet the definition of a TDA intervention (*n =* 16), unsuitable population (*n =* 4), unsuitable study focus area (*n =* 6), ineligible study design (*n =* 14), cross-referenced article (*n =* 8), unsuitable background interventions (*n =* 2), unsuitable comparison (*n =* 1), or because a full report could not be retrieved (e.g., abstract, protocol, or other document and ongoing studies) (*n =* 11). All full-text studies that did not meet eligibility criteria are listed in Supplemental Table 2, together with the main reasons for exclusion.

Five studies met the criteria for inclusion: two cluster-randomized controlled trials (cRCTs) conducted in Uganda^19^^,^^20^ and Kenya,^21^ one controlled before-after study conducted in Ghana,^22^ and two uncontrolled before-after studies conducted in Sri Lanka^23^ and Greece.^24^ Furthermore, two articles were assessed for contextual factors, one for resource use,^25^ and one for acceptability.^26^ Descriptive characteristics of the five included studies are summarized in Table 1 and described in detail in Supplemental Table 3.

**Table 1:** Characteristics of included studies

Study	Location	Year(s)	Study Design	Intervention	Outcomes Reported
Staedke et al.^19^ Rehman et al.^20^	Eastern Uganda	2014	cRCT	Target population: schoolchildren Intervention: IPT Comparator: standard of care Drug: DHA/PPQ Rounds: six, monthly	Prevalence of infection at the community level (1–4 months after intervention) SAEs (measured during delivery of intervention, month 0) Prevalence of infection among those targeted (last month of intervention, month 0)
Clarke et al.^21^	Western Kenya	2005–2006	cRCT	Target population: schoolchildren Intervention: IPT Comparator: placebo Drug: SP/AQ Rounds: three, every 4 months	Prevalence of infection among those targeted (1–2 months after intervention) SAEs and AEs (within 28 days of any treatment)
Opoku et al.^22^	Upper east region Ghana	2011	cBF	Target population: schoolchildren Intervention: IPT Comparator: treatment with anthelmintic Drug: AL Rounds: three, every 3 months	Prevalence of infection among those targeted (1 month after intervention)
Marasinghe et al.^23^	Southwestern Sri Lanka	2018	uBF	Target population: migrant workers Intervention: mass radical treatment Comparator: not applicable Drug: chloroquine + primaquine (low dose) Rounds: single round	Prevalence of infection among those targeted (1–5 months after intervention) SAEs (1–5 months after intervention)
Tseroni et al.^24^	Southern Greece	2013–2014	uBF	Target population: migrant workers Intervention: mass radical treatment Comparator: not applicable Drug: chloroquine + primaquine (high dose) Rounds: one, every year	Prevalence of infection among those targeted (1–6 months after intervention) AEs (1–6 months after intervention)

AE = adverse event; AL = artemether-lumefantrine; cBF = controlled before-after; cRCT = cluster-randomized controlled trial; DHA/PPQ = dihydroartemisinin-piperaquine; IPT = intermittent preventive treatment; SAE = serious adverse event; SP/AQ = sulfadoxine-pyrimethamine + amodiaquine; uBF = uncontrolled before-after.

The intervention in three of the studies^19^^–^^22^ targeted school-aged children, whereas migrant workers were targeted in the remaining two studies.^23^^,^^24^ The interventions implemented among schoolchildren were described by the researchers as IPT rather than TDA, but the description of the intervention met the definition included in the PICO model. In the studies targeting school-aged children, the number and frequency of rounds of TDA ranged from six rounds at monthly intervals^19^^,^^20^ to three rounds every 3^22^ or 4 months,^21^ and the comparison arms were standard of care,^19^^,^^20^ placebo,^21^ or anti-helminthic medicines.^22^ The drugs used in the studies targeting school-aged children were dihydroartemisinin-piperaquine,^19^^,^^20^ sulfadoxine-pyrimethamine with amodiaquine,^21^ and artemether-lumefantrine.^22^ The two studies targeting migrants administered a single round of TDA consisting of medicines to target both blood-stage and liver-stage parasites (chloroquine in combination with low-dose primaquine in Sri Lanka and high-dose primaquine in Greece).^23^^,^^24^

### Assessment of quality.

Assessments of risk of bias for the outcomes assessed in the two cRCTs^19^^–^^21^ are summarized in Supplemental Figures 1 and 2. The risk of bias for all outcomes was found to be low across all assessed domains except for missing outcome data. For AEs, Staedke et al.^19^ was rated as high risk, as the outcome was not measured in the comparison arm. Domains were assessed as described by Higgins et al.^27^

Supplemental Figure 3 summarizes the risk of bias assessment for the controlled before-after study.^22^ All domains were rated as low risk of bias except for biases due to confounding and deviations from intended interventions. The former was rated as moderate because the study reported crude prevalence proportions based on the number of study participants without accounting for other potential influences to study outcomes. The latter was also rated as moderate because the planned study design had to be changed from a randomized controlled trial to a controlled before-after design owing to limitations with funding constraints and expansion to accommodate other thematic areas. Because there was no description of blinding of the outcome assessment, we did not have information to assess bias in the measurement of outcomes. Domains were assessed as described by Sterne et al.^28^

The two uncontrolled before-after studies^23^^,^^24^ were rated as critical overall risk of bias owing to the inherent biases associated with the study design and thus were not assessed further using a risk-of-bias tool.

### Effect of interventions.

Staedke et al.^19^ reported data on the prevalence of infection at the population level 1 to 4 months after intervention measured through cross-sectional community surveys in randomly selected households. The study reported a lower prevalence in the intervention clusters than in the control clusters, 19% versus 23% (adjusted RR: 0.85, 95% CI: 0.73–1.00; the RR was adjusted for baseline parasite prevalence, sex, individual bed net use, school type, subcounty, socioeconomic status, eaves and window screening status, and latitude). The cRCT included 8,922 participants and was evaluated as having high-certainty evidence.

That same trial by Staedke et al.^19^ monitored serious AEs (SAEs) in all participants in the intervention arm during the delivery of the intervention. There were 17 SAEs reported, two considered as possibly related to the drug administration intervention. Adverse events were not monitored in the control arm, so an RR was not estimable, and the study was evaluated as having very low–certainty evidence. Clarke et al.^21^ monitored SAEs and AEs for 3 days after each treatment and a further 28 days thereafter using a passive surveillance system, reporting a total of 74 AEs in the intervention group (19 serious, 6 moderate, and 49 mild) and 44 in the control group (4 serious, 7 moderate, and 33 mild). As a result, the SAEs were evaluated as RR: 4.19, 95% CI: 1.43–12.31, low-certainty evidence and the AEs as RR: 1.48, 95% CI: 0.12–18.02, low-certainty evidence. Marasinghe et al.^23^ monitored SAEs for 5 months after the intervention, and no serious events were reported during or after the treatment (RR not estimable, very low–certainty evidence), whereas Tseroni et al.^24^ recorded AEs for 6 months after the intervention in 397 of the 1,094 treated individuals and the majority were classified as minor. A single case of primaquine-induced hemolysis was recorded in a person with a false glucose-6-phosphate-dehydrogenase test result. The total number of AEs reported was 688 (RR not estimable, very low–certainty evidence).

**Figure 2.**
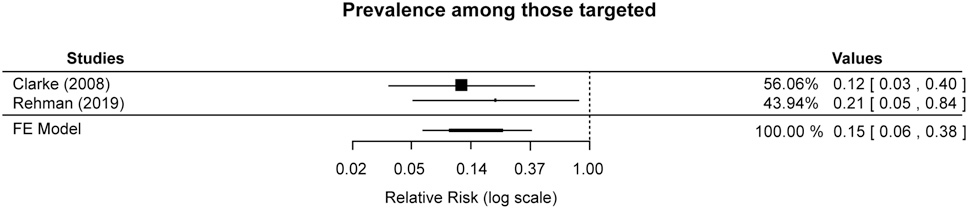
Effect of targeted drug administration versus no targeted drug administration on prevalence among the targeted population (cluster-randomized controlled trials). FE = fixed-effects.

**Figure 3.**
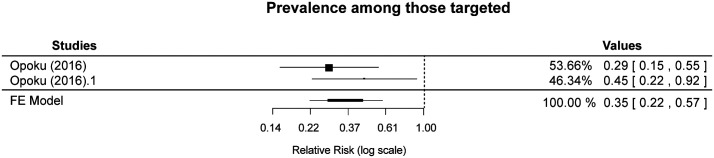
Effect of targeted drug administration versus no targeted drug administration on prevalence among the targeted population (controlled before-after study). FE = fixed-effects.

The impact of TDA on the prevalence of infection among the targeted populations was reported in Clarke et al.^21^ at 1 to 2 months after intervention and during the last month of the intervention in Rehman et al.^20^ Both studies reported a lower prevalence in the intervention arm than in the control arm. When the effects were combined in a meta-analysis, the pooled effect showed a reduction in prevalence among those targeted by the drug administration compared with no intervention (RR: 0.15, 95% CI: 0.06–0.38, *P*-value <0.001, moderate-certainty evidence; Figure 2). Opoku et al.^22^ reported on the prevalence of infection among the targeted population in the month after the intervention in the two intervention arms compared with the control arm. The unadjusted pooled effect showed a reduction in prevalence among those targeted by the drug administration intervention compared with no intervention (unadjusted RR: 0.35, 95% CI: 0.22–0.57, low-certainty evidence; Figure 3). The two uncontrolled before-after studies also reported on the prevalence of infection among the targeted population. Marasinghe et al.^23^ was conducted in Sri Lanka, which had been free from indigenous cases of malaria for the previous 6 years until the first introduced case was reported among a group of migrant workers, and the Tseroni et al.^24^ study was conducted in Greece, in an area where malaria had previously been eliminated. Both studies reported no malaria cases during the follow-up periods of 5 months after intervention^23^ and at least 6 months after intervention^24^ (very low–certainty evidence).

The CoEs according to the GRADE criteria are presented in Table 2. The certainty was high for the impact of TDA on the prevalence of malaria at the population level and moderate for the prevalence of malaria among those targeted by the intervention, but it was rated as very low or low for outcomes related to AEs or SAEs.

**Table 2:** GRADE summary of findings

Should TDA vs. No TDA be Used for Reducing Malaria Transmission?						
TDA Compared with No TDA for Reducing Malaria Transmission						
Outcome	Anticipated Absolute Effects* (95% CI)		Relative Effect (95% CI)	Number of Participants (studies)	Certainty	Notes
	Risk with No TDA	Risk with TDA				
Prevalence of malaria infection (community level), 1–4 months after intervention	219 per 1,000	186 per 1,000 (160–219)	RR: 0.85 (0.73–1.00)	8,922 (1 RCT)^19^	++++ High	TDA results in little to no difference in prevalence of malaria infection at the community level
Serious adverse events, randomized trials	0 per 1,000	0 per 1,000 (0–0)	Not estimable	10,079 (1 RCT)^19^	+OOO Very low^†^^‡^	Evidence is very uncertain about the effect of TDA on serious adverse events
Serious adverse events, randomized trials	2 per 1,000	7 per 1,000 (2–21)	RR: 4.19 (1.43–12.31)	4,916 (1 RCT)^21^^§^	++OO Low^‖^	TDA may result in little to no difference in serious adverse events
Serious adverse events, nonrandomized trials	0 per 1,000	0 per 1,000 (0–0)	Not estimable	31 (one observational study)^23^	+OOO Very low^¶^^#^	Evidence is very uncertain about the effect of TDA on serious adverse events
Adverse events, randomized trials	19 per 1,000	28 per 1,000 (2–344)	RR: 1.48 (0.12–18.02)	4,916 (1 RCT)^21^^§^	++OO Low^‖^	TDA may result in a slight increase in adverse events
Adverse events, nonrandomized trials	0 per 1,000	0 per 1,000 (0–0)	Not estimable	1,094 (one observational study)^24^	+OOO Very low^¶^	Evidence is very uncertain about the effect of TDA on adverse events
Prevalence among those targeted by the intervention,* 0–2 months after intervention	406 per 1,000	61 per 1,000 (24–154)	RR: 0.15 (0.06–0.38)	5,970 (two RCTs)^20^^,^^21^	+++O Moderate**	TDA likely/probably results in a large reduction in prevalence among those targeted by the intervention
Prevalence among those targeted by the intervention,* nonrandomized trials, 1 month after intervention	315 per 1,000	110 per 1,000 (69–180)	RR: 0.35 (0.22–0.57)	348 (one observational study)^22^^††^	++OO Low**^‡‡^	TDA may result in a reduction in prevalence among those targeted by the intervention
Prevalence among those targeted by the intervention,* nonrandomized trials, 1–6 months after intervention	Both studies reported no malaria cases during the follow-up periods			(Two observational studies)^23^^,^^24^	+OOO Very low^¶^^#^**	The evidence is very uncertain about the effect of TDA on prevalence among those targeted by the intervention

GRADE = Grading of Recommendations Assessment, Development and Evaluation; RCT = randomized controlled trial; RR = risk ratio; TDA = targeted drug administration. This table appears in color at www.ajtmh.org.

* Used as an intermediate outcome for prevalence at the community level.

^†^ Outcome was collected in intervention arm only.

^‡^ Unable to calculate effect measure in the absence of control measures.

^§^ Not corrected for unit of analysis error because of extremely wide CIs.

^‖^ Wide CIs.

^¶^ Critical overall risk of bias due to inherent biases associated with study design.

^#^ Few patients and few events.

** Used as a surrogate for prevalence of infection at the community level.

^††^ Study with two intervention arms. Both arms have been pooled and compared with the control.

^‡‡^ Moderate risk of bias due to confounding, bias due to deviations from intended interventions, and no information about bias in the measurement of outcomes.

### Contextual factors.

Data on resource use for TDA was abstracted from Temperley et al.^25^ on costs and cost effectiveness. This was linked to the school-based delivery of IPT in western Kenya reported by Clarke et al.^21^ The authors concluded that IPT administered by teachers compared favorably with the estimated costs of other interventions, such as insecticide-treated nets or indoor residual spraying, with the largest cost components being the drugs and teacher training. Acceptability of IPT for malaria in schoolchildren was reported in one qualitative research study, Matangila et al.,^26^ which aimed to understand the perceptions and experiences of parents and teachers related to IPT after a clinical trial in two schools of Democratic Republic of the Congo. The acceptability of TDA was affected by whether trained clinical staff administered the medicine, but there was concern that medicines were provided without parasitological diagnosis.

## DISCUSSION

Few studies contributed data to assess the benefits and harms of TDA, and overall, the CoE was low. Based on results from a single cRCT, TDA resulted in little to no difference in the prevalence of malaria infection at the population level. However, pooled results from two cRCTs suggest that TDA likely resulted in a large reduction in prevalence among those targeted by the intervention. The studies included in this review provided intermittent chemoprevention to schoolchildren in moderate- to high-transmission settings, and the effect of this intervention on transmission depends on the proportion of the reservoir of infection represented by this targeted population. It is quite plausible that chemoprevention may benefit the group receiving the antimalarial medicine without reducing transmission at the population level if the risk group does not represent a sufficient proportion of the infectious reservoir. Other factors that could influence the effect of TDA on malaria transmission include variations in risk and exposure levels between the targeted population and the general population, as well as their interactions and connectivity. Moreover, the type of antimalarial drug administered, number and frequency of rounds, background interventions, and coverage achieved are factors that may also affect the impact of the intervention and should also be considered. Two studies contributed data on contextual factors, suggesting that TDA is a cost-effective intervention (for reductions in the targeted population) when delivered at schools by teachers and emphasizing the importance of addressing any misperceptions about the intervention (e.g., safety of the drugs used) to increase ownership and acceptability of the community.

The principle behind targeted strategies is the ability to identify and administer medicines to groups who have the highest rates of exposure to infected mosquitoes and account for the vast majority of the reservoir of infection in an area. TDA could be a good strategy for program interventions that aim to target specific populations in transmission foci or areas where migrants may import infections. In southeast Asia, for example, forested areas are one of the remaining foci of malaria transmission, and forest-goers are groups who are highly exposed to infected mosquitoes for limited periods of time. Providing chemoprevention to this group could reduce transmission while protecting forest workers from the consequences of malaria infection. Likewise, migrant laborers or refugees originating from a malaria endemic area could benefit from this intervention if they are more likely to carry malaria parasites than the general population.

Other forms of chemoprevention in groups such as pregnant women, infants, and young children were excluded from this review as the objective of these interventions (including IPT in pregnancy and seasonal malaria chemoprevention) is to reduce the risk of severe disease or severe consequences of malaria infection rather than to reduce transmission. Moreover, these are interventions already recommended by the WHO.^29^ In moderate- to high-transmission settings, chemoprevention reduces the burden of disease, whereas TDA is considered more appropriate in areas approaching elimination. Indeed, after studying the results of this systematic review, the WHO Guideline Development Group (GDG) decided that TDA would be most relevant in low- to very low–transmission settings and subsequently revised the PICO question for the purposes of WHO guideline development. Therefore, the WHO GDG considered only the two studies conducted in post-elimination settings^23^^,^^24^ as direct evidence of the impact of TDA.^5^ Even though the studies targeting schoolchildren in moderate- to high-transmission settings^19^^–^^22^ were included in this review, as the description of the intervention met the definition included in the PICO model, it is worth noting that their overall goal was to reduce the burden of disease, with less focus on reducing transmission. Hence, to specifically assess studies that aimed to reduce transmission and that targeted populations considered to be important reservoirs of infection, the PICO questions of future reviews of targeted strategies (including targeted testing and treatment)^30^ could either be restricted to very low– to low-transmission settings or to populations proven to host the vast majority of the reservoir of infection.

Despite the overall low quality of evidence, the WHO provided a conditional recommendation for TDA, particularly in post-elimination settings to prevent reestablishment of transmission.^5^ However, further evidence is needed on the impact of TDA in very low– to low-transmission areas to strengthen this recommendation.

## Supplemental Materials

10.4269/ajtmh.22-0754 Supplemental Materials
