# Supplementary material for: Targeted Drug Administration to Reduce Malaria Transmission: A Systematic Review and Meta-Analysis
Source: Am J Trop Med Hyg. 2024 Jan 23;110(4 Suppl):65–72. doi: 10.4269/ajtmh.22-0754 (PMC10993794; doi:10.4269/ajtmh.22-0754)
Supplement: Supplemental Materials [file tpmd220754.SD1.pdf]

SUPPLEMENTAL DATA

Targeted Drug Administration to Reduce Malaria Transmission: A Systematic Review and Meta-Analysis

Maria Tusell,[1*] Elisabet Martí Coma-Cros,[1] Beena Bhamani,[1] Vita Mithi,[2,3,4] Elisa Serra-Casas,[1] Nana Aba Williams,[1] Kim A. Lindblade,[5] Koya C. Allen[1]

[1] Barcelona Institute for Global Health (ISGlobal), Hospital Clínic - Universitat de Barcelona, Barcelona, Spain

[2] Armref Data for Action in Public Health Research Consultancy, Mzuzu, Malawi

[3] Society for Research on Nicotine and Tobacco-Genetics and Omics Network, Madison, USA

[4] Leaders of Africa Institute, Baltimore, USA

[5] Global Malaria Program, World Health Organization, Geneva, Switzerland

[*]Address correspondence to Maria Tusell, Barcelona Institute for Global Health (ISGlobal), Hospital Clínic - Universitat de Barcelona, Carrer Rosselló 171 Entl. 2a, Barcelona 08036. Email: maria.tusell@isglobal.org

Supplemental Table 1. Search strategy

1	*malaria/
2	exp malaria, falciparum/ or exp malaria, vivax/
3	malaria ovale.mp. or Plasmodium ovale/
4	plasmodium malariae.mp. or Plasmodium malariae/
5	1 or 2 or 3 or 4
6	Antimalarials/
7	Disease Eradication/ or elimination.tw
8	(tailored adj2 (intervention* or treatment* or strateg* or administration)).tw
9	(relapse adj2 prevention).mp
10	Presumptive adj2 (treatment or therapy).tw
11	focal adj2 (drug administration).tw or “focal MDA”.tw
12	targeted adj2 (intervention* or treatment or strateg* or administration).tw
13	6 or 7 or 8 or 9 or 10 or 11 or 12
14	(“Forest-goers “ or Forests/ or Vulnerable or Worksites or Farm* OR plantation* or mining or miner* or Miners/ or laborers or cultivators or military or “armed forces” or Military Personnel/ or “peace-keepers” or agricultural)
15	“high risk population*”.mp. or Risk Factors/
16	“special populations”.tw
17	“high exposure”.tw or “highly exposed”.tw or “high transmission”.tw or hotspot*.tw
18	14 or 15 or 16 or 17
19	5 and 13 and 18

Supplemental Table 2. List of studies excluded after full review and primary reasons for exclusion

Study	Reference	Primary reasons for exclusion
**Ahmed 2019**	1	Unsuitable population
**Ambroise- Thomas 2001**	2	Not a TDA study: Prophylaxis; Report could not be retrieved
**Anonymous 1948**	3	Report could not be retrieved
**Anonymous 2016**	4	Study focus area
**Armauer 2020**	5	Not a TDA study: Reactive TDA
**Barger 2009**	6	Study design: individual randomization
**Bennett 2020**	7	Not a TDA study: Reactive TDA; Cross-referenced study: Finn (2020), Silumbe (2020)
**Betuela 2012**	8	Study design: individual randomization
**Bigira 2014**	9	Study design: individual randomization
**Boivin 2016**	10	Cross-referenced article: Bigira 2014
**Boivin 2014**	11	Cross-referenced article: Bigira 2014; Abstract only/could not be retrieved
**Breeveld 2012**	12	Study focus area: review
**Buchwald 2018**	13	Abstract only/could not be retrieved
**Chandramohan 2005**	14	Not a TDA study: other strategy (IPTi)
**Clarke 2012**	15	Abstract only/could not be retrieved
**Clarke 2017**	16	Imbalance of background interventions: teacher-led participatory malaria prevention education implemented only in intervention arm
**Cohee 2018**	17	Imbalance of background interventions: peer education program implemented only in intervention arm
**Dantzer 2017**	18	Abstract only/could not be retrieved; Not a TDA study
**Dicko 2008**	19	Not a TDA study: expanded SMC
**Dobaño 2019**	20	Not the correct outcomes reported
**Edstein 2001**	21	Study design: individual randomization
**Finn 2020**	22	Cross referenced article: Bennett (2020), Silumbe (2020); Not a TDA study: Reactive TDA
**Grobusch 2007**	23	Not a TDA study: other strategy (IPTi)
**Guyant 2015**	24	Study focus area
**Hill 2016**	25	Unsuitable population
**Houel 1954**	26	Not a TDA study: Prophylaxis
**Hoyt 2018**	27	Unsuitable population
**Hygiene 2008**	28	Study design: individual randomization
**Huch 2018**	29	Abstract only/could not be retrieved
**Janssens 1955**	30	Report could not be retrieved
**Jongdeepaisal 2021**	31	Not a TDA study: Prophylaxis
**Jongdeepaisal 2022**	32	Not a TDA study: Prophylaxis
**Jongdeepaisal 2022b**	33	Not a TDA study: Prophylaxis
**Kunkel 2021**	34	Ongoing, results TDA not reported
**Landier 2016**	35	Protocol only; Unsuitable population: not targeted to high-risk groups, targeted to higher incidence hotspots; Cross-referenced article: Sahan (2017)
**Liljander 2010**	36	Not a TDA study: SMC
**Lon 2015**	37	Cross-reference: Manning 2018; Abstract only
**Lwin 2012**	38	Study design: individual randomization

**Makenga 2020**	[39]	Protocol only; Cross-referenced study: National 2019
**Manning 2018**	[40]	Inappropriate comparison; Protocol only; Cross-referenced study: Lon (2015)
**Manore 2019**	[41]	Study focus area
**Maude 2021**	[42]	Study design: individual randomization; Protocol
**Matangila 2017**	[43]	Study design: individual randomization
**Miller 1955**	[44]	Study design: controlled before-after, but only 1 site per arm
**MSF 2020**	[45]	Ongoing study with no results available yet; Abstract only
**Nankabirwa 2010**	[46]	Cross-referenced study: Hygiene 2008
**Nankabirwa 2014**	[47]	Study design: individual randomization
**National 2019**	[48]	Protocol only; Cross-referenced study: Makenga 2020
**Nikolov 2017**	[49]	Unsuitable population: not targeted to high-risk groups; Abstract only
**Rohner 2010**	[50]	Study design: individual randomization
**Sahan 2017**	[51]	Unsuitable population: not targeted to high-risk groups, targeted to higher incidence hotspots; Cross-referenced article: Landier (2016)
**Silumbe 2020**	[52]	Not a TDA study: Reactive TDA; Cross-referenced article: Finn (2020), Bennett (2020)
**Son 2017**	[53]	Study design: individual randomization
**Steketee 1996**	[54]	Not a TDA study: IPTp
**Stingl 2004**	[55]	Report could not be retrieved
**Thera 2018**	[56]	Study design: individual randomization
**Tuck 2005**	[57]	Not a TDA study
**UCSF 2019**	[58]	Ongoing study with no results available yet
**Villegas 2010**	[59]	Not a TDA study; Abstract only
**von Seidlein 2019**	[60]	Study focus area: review
**Wen 2016**	[61]	Not a TDA study
**Xu 2021**	[62]	Study focus area: review

Supplemental Figure 1. Risk of bias summary for the outcomes assessed in the cRCTs

Study	Outcome	D1a	D1b	D2	D3	D4	D5	Overall
Staedke 2018	Prevalence community	+	+	+	+	+	+	+
Staedke 2018	SAEs	+	+	+	-	+	+	-
Rehman 2019	Prevalence targeted	+	+	+	+	+	+	+
Clarke 2008	AEs (including SAEs)	+	+	+	+	+	+	+
Clarke 2008	Prevalence targeted	+	+	+	+	+	+	+

+ Low risk
! Some concerns
- High risk

Supplemental Figure 2. Risk of bias summary for the cRCTs by percentage

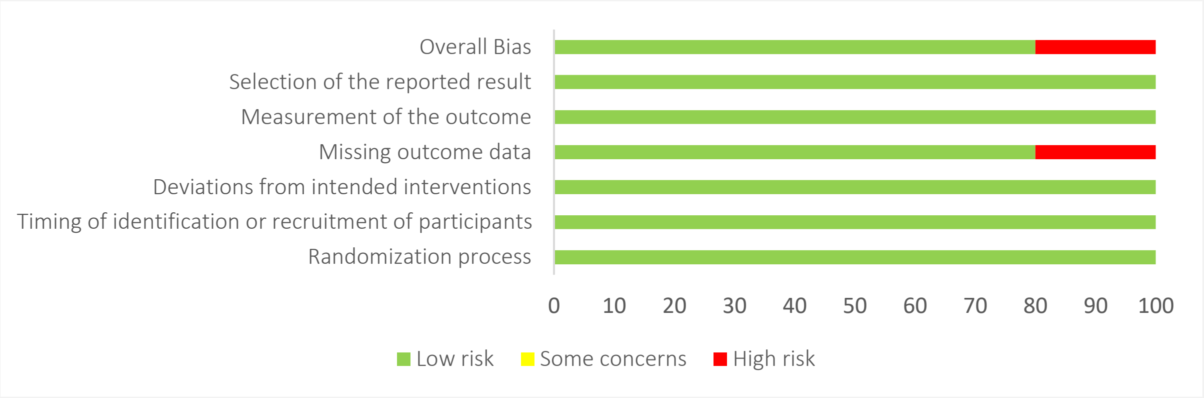

Supplemental Figure 3. Risk of bias summary for the controlled before-after study

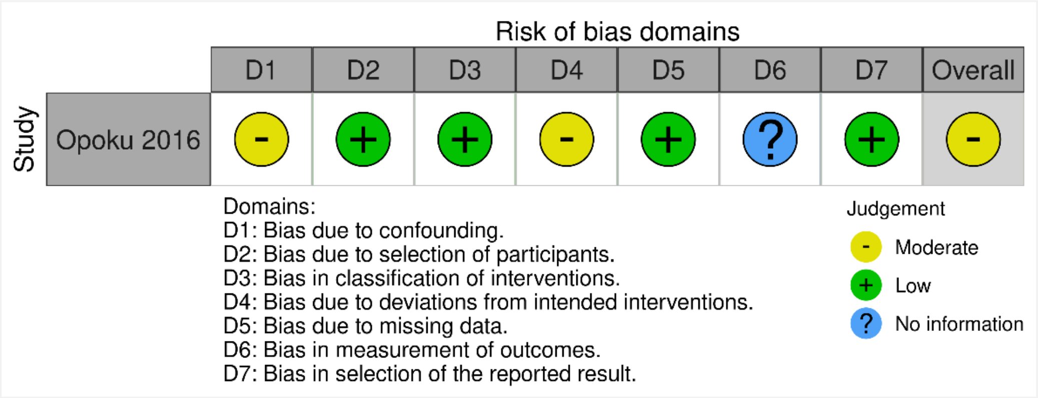

Supplemental Table 3. Data extracted from included studies

Staedke 2018[63] and Rehman 2019[64]

**Study characteristics**	
METHODS	
**Study dates**	2014-2015
**Location**	Jinja District, eastern Uganda
**Peak transmission season**	Perennial transmission
**Baseline transmission intensity**	Moderate, baseline cluster parasite prevalence (median) was 25.4% in the intervention group and 25.2% in the control group
**Parasite species**	*Plasmodium falciparum*
**Vector species**	*Anopheles gambiae*, *Anopheles funestus*, *Anopheles arabiensis*
**Study design**	Cluster-randomized controlled trial
**Statistical power calculation**	Assuming significance of 5% and power of 80%, authors calculated that 105 individuals per cluster (minimum total 8820) would allow detection of a relative reduction in parasite prevalence of 29%, based on 21% prevalence in the control group
**Clusters**	Unit of randomization: Primary school + 100 closest households<br>Features of the clusters: Buffer areas, restricted randomization<br>Number of clusters selected: 84<br>Number of clusters analyzed: 84<br>Average cluster size: 35 households, 119 participants (median)
PARTICIPANTS	
**Targeted population**	Total: 42 351*<br>Intervention: 23 280 students in intervention group eligible for IPT, 3030 households screened for baseline community survey, 2097 households approached for final community survey<br>Comparison: 2957 households screened for baseline community survey, 2160 households approached for final community survey
**Participant characteristics**	School-aged children, 5 to 20 years
INTERVENTION	
**Intervention**	Intermittent preventive treatment
**Comparator**	Standard of care
**Background interventions**	No background interventions described
**Drug and manufacturer**	Dihydroartemisinin- piperaquine, Duo-Cotexcin tablets (Beijing Holley-Cotec Pharmaceuticals, Beijing, China)
**Dosage**	40 mg dihydroartemisinin and 320 mg piperaquine
**Number of rounds per season or year**	6
**Treatment interval**	Monthly
**Duration of the intervention**	6 months
**Treatment adherence**	Round 1: 2680 (12%), Round 2: 3287 (14%), Round 3: 3601 (15%), Round 4: 8154 (36%), Round 5: 8628 (37%), Round 6: 6714 (29%)
OUTCOMES	

**Prevalence of infection at the community level**	Measurement: Cross-sectional community surveys in randomly-selected households, measured by microscopy<br>Timepoints: 1-4 months post-intervention<br>Sample size: 4455 (intervention); 4467 (control)
**Serious Adverse Events**	All intervention participants were monitored for serious adverse events, and a subset of participants selected by convenience sampling for cardiac monitoring. 17 serious adverse events were reported, including two deaths (one from a road traffic accident and one from a tetanus infection, both unrelated to the drug). Among the remaining 15 serious adverse events, two (an allergic skin reaction and a case of weakness and loss of consciousness after severe abdominal pain that was attributed to hypoglycemia) were judged to be possibly related to the drug.
**Prevalence of infection among those targeted by the intervention**	Measurement: Cross-sectional survey of randomly-selected children from each participating school<br>Timepoints: Toward the end of the intervention, month 0<br>Sample size: 546 (intervention); 546 (control)

*Intervention: 23 280 students in intervention group eligible for IPT + 5037 residents screened for baseline community survey + 4477 residents screened for final community survey; Control: 5038 residents screened for baseline community survey + 4519 residents screened for final community survey.

**Risk of bias**		
Outcome: **Prevalence of infection at the community level**		
**Domain**	**Author's judgement**	**Justification**
**D1a: Randomization process**	Low risk	"Used restricted randomization to ensure balance across clusters for geographical location by subcounty and school type (public or private)."
**D1b: Timing of identification or recruitment of participants**	Low risk	"Study personnel enrolled primary schools after cluster randomization without masking of study group allocation. An information sheet described the study and verbal consent to participate was obtained after randomization". Baseline characteristics were similar across study groups.
**D2: Deviations from the intended interventions**	Low risk	Nothing to indicate that the intervention had any deviations from its intended format based on the trial context.
**D3: Missing outcome data**	Low risk	Data reported for all clusters. "Sample size calculations for the final community survey were informed by data collected in the baseline survey."
**D4: Measurement of the outcome**	Low risk	Outcomes "read by experienced laboratory technologists who were unaware of study group assignments."
**D5: Selection of the reported result**	Low risk	Reported primary and secondary outcomes are the same as reported on clinicaltrials.gov.

**Risk of bias**		
Outcome: **Serious Adverse Events**		
**Domain**	**Author's judgement**	**Justification**

**D1a: Randomization process**	Low risk	"Used restricted randomization to ensure balance across clusters for geographical location by subcounty and school type (public or private)."
**D1b: Timing of identification or recruitment of participants**	Low risk	Not applicable based on study design. "Study personnel enrolled primary schools after cluster randomization without masking of study group allocation. An information sheet described the study and verbal consent to participate was obtained after randomization". Baseline characteristics were similar across study groups.
**D2: Deviations from the intended interventions**	Low risk	Nothing to indicate that the intervention had any deviations from its intended format based on the trial context.
**D3: Missing outcome data**	High risk	Adverse events were not monitored in the control arm.
**D4: Measurement of the outcome**	Low risk	"All intervention participants were monitored for serious adverse events, and a subset of participants selected by convenience sampling for cardiac monitoring."
**D5: Selection of the reported result**	Low risk	Reported primary and secondary outcomes are the same as reported on clinicaltrials.gov.

**Risk of bias**		
Outcome: **Prevalence of infection among those targeted by the intervention**		
**Domain**	**Author's judgement**	**Justification**
**D1a: Randomization process**	Low risk	"Used restricted randomization to ensure balance across clusters for geographical location by subcounty and school type (public or private)."
**D1b: Timing of identification or recruitment of participants**	Low risk	Not applicable based on study design. "Study personnel enrolled primary schools after cluster randomization without masking of study group allocation. An information sheet described the study and verbal consent to participate was obtained after randomization". Baseline characteristics were similar across study groups.
**D2: Deviations from the intended interventions**	Low risk	Nothing to indicate that the intervention had any deviations from its intended format based on the trial context.
**D3: Missing outcome data**	Low risk	Data reported for all clusters. "Sample size was determined for the trial's secondary outcome, smear positive microscopy among school children, and was refined after results from the baseline survey were available."
**D4: Measurement of the outcome**	Low risk	Outcomes "read by experienced laboratory technologists who were unaware of study group assignments."
**D5: Selection of the reported result**	Low risk	Reported primary and secondary outcomes are the same as reported on clinicaltrials.gov.

33 ***Clarke 2008***[65]

Study characteristics	
METHODS	
**Study dates**	2005-2006
**Location**	Bondo District, western Kenya
**Peak transmission season**	March-May and November-December
**Baseline transmission intensity**	High, baseline parasite prevalence was 41% in the intervention group and 42% in the control group
**Parasite species**	*Plasmodium falciparum*
**Vector species**	Not described
**Study design**	Cluster-randomized controlled trial
**Statistical power calculation**	80% power to detect a 30% reduction in the prevalence of anaemia in the intervention group compared with placebo at 5% significance
**Clusters**	Unit of randomization: Primary school<br>Features of the clusters: Stratification<br>Number of clusters selected: 30<br>Number of clusters analyzed: 30<br>Average cluster size: 225
PARTICIPANTS	
**Targeted population**	Total: 6758<br>Intervention: 3535<br>Comparison: 3223
**Participant characteristics**	School-aged children, 5 to 18 years
INTERVENTION	
**Intervention**	Intermittent preventive treatment
**Comparator**	Placebo
**Background interventions**	Mass treatment with anthelmintics
**Drug and manufacturer**	Sulfadoxine-pyrimethamine + amodiaquine, Cosmos Limited
**Dosage**	Sulfadoxine-pyrimethamine: single dose given over one day; amodiaquine: 3 daily doses over 3 days. Dosage given according to age.
**Number of rounds per season or year**	3
**Treatment interval**	Every 4 months
**Duration of the intervention**	1 year
**Treatment adherence**	1070 (41%) received treatment on all 9 days
OUTCOMES	
**Serious Adverse Events**	Adverse events were monitored by the study team for 3 days after each treatment, and a further 28 days thereafter using a passive surveillance system in schools and local health centres. 23 serious adverse events were reported within 28 days of treatment, 19 in the intervention group (of which three were judged to be possibly associated with treatment), and 4 in the control group.

**Adverse Events**	Adverse events were monitored by the study team for 3 days after each treatment, and a further 28 days thereafter using a passive surveillance system in schools and local health centres. A total of 74 adverse events (including serious, moderate and mild) were reported in the intervention arm, and 44 in the control arm.
**Prevalence of infection among those targeted by the intervention**	Measurement: Cross-sectional survey<br>Timepoints: Approximately 6 weeks (1-2 months) post-intervention<br>Sample size: 2584 (intervention); 2294 (control)

**Risk of bias**		
Outcome: **AEs (including SAEs)**		
**Domain**	**Author's judgement**	**Justification**
**D1a: Randomization process**	Low risk	"Schools were randomly allocated to one of six coded drug groups by use of block randomization according to a computer-generated random number list by an investigator blind to the drug group."
**D1b: Timing of identification or recruitment of participants**	Low risk	"Ten schools were randomly selected from each school-performance stratum, and within each stratum schools were randomly allocated to one of six coded drug groups by use of block randomization according to a computer generated random number list by an investigator blind to drug group."
**D2: Deviations from the intended interventions**	Low risk	"Active drugs and placebos were similar in size and shape, but differed in taste. To preserve blinding, staff responsible for measuring health or education outcomes were unaware of drug group allocation, and analysis was done by a statistician with no previous involvement in the trial."
**D3: Missing outcome data**	Low risk	"Adverse events were monitored by the study team for 3 days after each treatment, and a further 28 days thereafter using a passive surveillance system in schools and local health centres."
**D4: Measurement of the outcome**	Low risk	"Staff responsible for measuring health and education<br>outcomes were unaware of drug group allocation, and<br>analysis was done by a statistician with no previous involvement in the trial."
**D5: Selection of the reported result**	Low risk	"An independent data safety monitoring board monitored the trial and approved the analysis plan." Reported primary and secondary outcomes are the same as reported on clinicaltrials.gov.

**Risk of bias**
Outcome: **Prevalence of infection among those targeted by the intervention**

Domain	Author's judgement	Justification
**D1a: Randomization process**	Low risk	"Schools were randomly allocated to one of six coded drug groups by use of block randomization according to a computer-generated random number list by an investigator blind to the drug group."
**D1b: Timing of identification or recruitment of participants**	Low risk	"Ten schools were randomly selected from each school-performance stratum, and within each stratum schools were randomly allocated to one of six coded drug groups by use of block randomization according to a computer generated random number list by an investigator blind to drug group."
**D2: Deviations from the intended interventions**	Low risk	"Active drugs and placebos were similar in size and shape, but differed in taste. To preserve blinding, staff responsible for measuring health or education outcomes were unaware of drug group allocation, and analysis was done by a statistician with no previous involvement in the trial."
**D3: Missing outcome data**	Low risk	"73% of children enrolled were examined in the postintervention survey 12 months later. Numbers of children lost to follow-up was similar in intervention and control groups."
**D4: Measurement of the outcome**	Low risk	"Staff responsible for measuring health and education<br>outcomes were unaware of drug group allocation, and<br>analysis was done by a statistician with no previous involvement in the trial."
**D5: Selection of the reported result**	Low risk	"An independent data safety monitoring board monitored the trial and approved the analysis plan." Reported primary and secondary outcomes are the same as reported on clinicaltrials.gov.

Opoku 2016[66]

**Study characteristics**	
METHODS	
**Study dates**	2011-2012
**Location**	Kassena-Nankana Districts, Upper East Region, Ghana
**Peak transmission season**	May-November
**Baseline transmission intensity**	Moderate, prevalence of malaria parasitemia was 28.7% among the schoolchildren at baseline
**Parasite species**	*Plasmodium falciparum*
**Vector species**	*Anopheles gambiae, Anopheles funestus, Anopheles arabiensis*
**Study design**	Three-arm, open-label, before-after intervention study including a control arm

**Statistical power calculation**	No power calculation conducted; purposive sampling ensured a fair representation of the districts based on previous data on *Schistosoma haematobium* and malaria transmission
**Clusters**	Not applicable
PARTICIPANTS	
**Targeted population**	Total: 348<br>Intervention: 131 (arm 1), 90 (arm 2)<br>Comparison: 127
**Participant characteristics**	School-aged children, 6 to 15 years
INTERVENTION	
**Intervention**	Intermittent preventive treatment
**Comparator**	Mass treatment with anthelmintics
**Background interventions**	Mass treatment with anthelmintics
**Drug and manufacturer**	Artemether-lumefantrine
**Dosage**	20/120, standard 3-day schedule according to body weight
**Number of rounds per season or year**	3
**Treatment interval**	Every 3 months
**Duration of the intervention**	7 months
**Treatment adherence**	Not described
OUTCOMES	
**Prevalence of infection among those targeted by the intervention**	Measurement: Parasitemia determined by microscopy<br>Timepoints: 1 month post-intervention<br>Sample size: 131 (arm 1); 90 (arm 2); 63.5 (control)*

*Sample size in the control arm used for the analysis has been 63.5 instead of 127 to account for the two intervention arms compared to the control arm.

**Risk of bias**		
Outcome: **Prevalence of infection among those targeted by the intervention**		
**Domain**	**Author's judgement**	**Justification**
**D1: Bias due to confounding**	Moderate risk	Crude prevalence proportions based on the study participants without accounting for other potential influences to study outcomes
**D2: Bias due to selection of participants**	Low risk	All participants who would have been eligible for the target trial were included in the study; for each participant, start of follow up and start of intervention coincided
**D3: Bias in classification of interventions**	Low risk	Intervention status is well defined
**D4: Bias due to deviations from**	Moderate risk	"Because of limitations due to funding constrains, and expansion to accommodate other thematic areas, the study, which was initially planned as a

**intended interventions**		randomized controlled trial, had to be changed to a before-after design.”
**D5: Bias due to missing data**	Low risk	97% follow-up
**D6: Bias in measurement of outcomes**	Unclear	No description of blinding of outcome assessment
**D7: Bias in selection of the reported result**	Low risk	Reported primary and secondary outcomes are the same as reported on clinicaltrials.gov

Marasinghe 2020[67]

**Study characteristics**	
METHODS	
**Study dates**	2018
**Location**	Monaragala District, Southwestern Sri Lanka
**Peak transmission season**	Not applicable
**Baseline transmission intensity**	Zero
**Parasite species**	*Plasmodium vivax*
**Vector species**	*Anopheles culicifacies*
**Study design**	Uncontrolled before-after
**Statistical power calculation**	Not applicable
**Clusters**	Not applicable
PARTICIPANTS	
**Targeted population**	Total: 31<br>Intervention: 31<br>Comparison: not applicable
**Participant characteristics**	Migrant workers of Indian origin in a factory construction site
INTERVENTION	
**Intervention**	Mass radical treatment
**Comparator**	Not applicable
**Background interventions**	No background interventions described
**Drug and manufacturer**	Chloroquine and primaquine
**Dosage**	Chloroquine 25 mg/kg body weight (over three days) and primaquine low dose (0.25 mg/kg/day bodyweight for 14 days)
**Number of rounds per season or year**	Single round
**Treatment interval**	Not applicable
**Duration of the intervention**	6 months
**Treatment adherence**	All 31 individuals received the full course of chloroquine

	24 individuals received primaquine for 14 days, the others were excluded due to G6PD deficiency
OUTCOMES	
**Serious Adverse Events**	Participants were monitored daily for the occurrence of adverse events until completion of the treatment and all were followed up at monthly intervals for a period of five months. No major adverse effects were reported during or after the treatment.
**Prevalence of infection among those targeted by the intervention**	Measurement: Parasitemia determined by microscopy<br>Timepoints: 5 months post-intervention<br>Sample size: 31 (intervention)

**Risk of bias**
Outcome: **All outcomes**
Critical overall risk of bias due to the inherent biases associated with the study design

Tseroni 2015[68]

**Study characteristics**	
METHODS	
**Study dates**	2013-2014
**Location**	Evrotas, southern Greece
**Peak transmission season**	May-December
**Baseline transmission intensity**	In 2012, 10 locally acquired cases and 17 imported cases were recorded in the area in an elimination setting preventing malaria reintroduction
**Parasite species**	*Plasmodium vivax*
**Vector species**	*Anopheles sacharovi*
**Study design**	Uncontrolled before-after
**Statistical power calculation**	Not applicable
**Clusters**	Not applicable
PARTICIPANTS	
**Targeted population**	Total: 1270<br>Intervention: 1270<br>Comparison: not applicable
**Participant characteristics**	Migrant farm laborers living in the epicenter of the 2011 outbreak
INTERVENTION	
**Intervention**	Mass radical treatment
**Comparator**	Not applicable
**Background interventions**	Community education, active case detection, vector control (IRS and LLINs)
**Drug and manufacturer**	Chloroquine and primaquine
**Dosage**	Chloroquine in the form of bisphosphate salts at an initial dose of 1000mg (620mg base) followed by 500mg at 6, 24 and 48 hours. Primaquine tablets were administered for 14 days at a dose of 30mg base per day (high dose)

**Number of rounds per season or year**	Single round
**Treatment interval**	Not applicable
**Duration of the intervention**	Two years
**Treatment adherence**	87.3% individuals successfully completed the treatment
OUTCOMES	
**Adverse Events**	All adverse effects were systematically recorded in structured pharmacovigilance forms, and were promptly treated by the health professionals from the field team. 688 adverse events were recorded in 397 individuals, the vast majority minor, predominantly dizziness and headache for chloroquine (284 events) and abdominal pain (85 events) for primaquine. A single case of primaquine-induced hemolysis was recorded in a person whose initial G6PD test proved incorrect.
**Prevalence of infection among those targeted by the intervention**	Measurement: Weekly fever screening visits in the context of active case detection. A rapid diagnostic test for malaria was performed to all individuals with fever or who reported fever and/or other malaria compatible symptoms during the previous week and blood was drawn for blood smear and molecular diagnosis of malaria.<br>Timepoints: Follow-up at least six months<br>Sample size: 1094

**Risk of bias**
Outcome: **All outcomes**
Critical overall risk of bias due to the inherent biases associated with the study design

References

1. Ahmed R, et al., 2019. Efficacy and safety of intermittent preventive treatment and intermittent screening and treatment versus single screening and treatment with dihydroartemisinin–piperaquine for the control of malaria in pregnancy in Indonesia: a cluster-randomised, open-label, superiority trial. *The Lancet Infectious Diseases 19*: 973–987
2. Ambroise-Thomas P., 2001. [Antimalarial prophylaxis for the traveler or in the armed forces]. *Med Trop (Mars) 61*: 5–6
3. Anonymous., 1948. A fine conquest over malaria realized by the Pasteur Institute of Algiers. [French]. *Revue du Paludisme et de Medecine Tropicale*
4. Anon. Malaria control improves for vulnerable in Africa, but global progress off-track. Available at: https://www.who.int/news/item/13-12-2016-malaria-control-improves-for-vulnerable-in-africa-but-global-progress-off-track. Accessed
5. Anon. Evaluation of Targeted Mass Drug Administration for Malaria in Ethiopia - Full Text View - ClinicalTrials.gov. Available at: https://clinicaltrials.gov/ct2/show/NCT04241705. Accessed
6. Barger B, Maiga H, Traore OB, Tekete M, Tembine I, Dara A, Traore ZI, Gantt S, Doumbo OK, Djimde AA., 2009. Intermittent preventive treatment using artemisinin-based combination therapy reduces malaria morbidity among school-aged children in Mali. *Tropical Medicine & International Health 14*: 784–791
7. Bennett A, et al., 2020. A Longitudinal Cohort to Monitor Malaria Infection Incidence during Mass Drug Administration in Southern Province, Zambia. *Am J Trop Med Hyg 103*: 54–65
8. Betuela I, et al., 2012. Relapses Contribute Significantly to the Risk of Plasmodium vivax Infection and Disease in Papua New Guinean Children 1–5 Years of Age. *The Journal of Infectious Diseases 206*: 1771–1780
9. Bigira V, et al., 2014. Protective Efficacy and Safety of Three Antimalarial Regimens for the Prevention of Malaria in Young Ugandan Children: A Randomized Controlled Trial. *PLoS Med 11*: e1001689
10. Boivin MJ, et al., 2016. Malaria illness mediated by anaemia lessens cognitive development in younger Ugandan children. *Malaria Journal 15*: 210

11. Boivin M J, et al., 2014. Bouts of malaria illness as mediated by anemia diminishes cognitive development in very young Ugandan children. *American Journal of Tropical Medicine and Hygiene*

12. Breeveld FJ, Vreden SG, Grobusch MP., 2012. History of malaria research and its contribution to the malaria control success in Suriname: a review. *Malaria Journal 11*: 95

13. Buchwald., 2018. School-based antimalaria interventions efficiently reduce community-level plasmodium falciparum prevalence in a high-transmission setting: A mathematical modeling study (abstract). *American Journal of Tropical Medicine and Hygiene*

14. Chandramohan D, et al., 2005. Cluster randomised trial of intermittent preventive treatment for malaria in infants in area of high, seasonal transmission in Ghana. *BMJ 331*: 727–733

15. Sian E. Clarke., 2012. A New Approach for Malaria Control in Schools: Results of a Randomized Trial of Intermittent Parasite Clearance. *American Journal of Tropical Medicine and Hygiene*

16. Clarke SE, et al., 2017. Impact of a malaria intervention package in schools on *Plasmodium* infection, anaemia and cognitive function in schoolchildren in Mali: a pragmatic cluster-randomised trial. *BMJ Glob Health 2*: e000182

17. Cohee LM, Chilombe M, Ngwira A, Jemu SK, Mathanga DP, Laufer MK., 2018. Pilot Study of the Addition of Mass Treatment for Malaria to Existing School-Based Programs to Treat Neglected Tropical Diseases. *The American Journal of Tropical Medicine and Hygiene 98*: 95–99

18. Dantzer E ; Lover A A; Hongvanthong B ; Chindavongsa K ; Welty S ; Reza T ; Nanthana V ; Hocini S ; Bennett A ;, 2017. Formative assessment to understand and target high-risk populations for malaria infection, champasak province, lao pdr. *American Journal of Tropical Medicine and Hygiene*

19. Dicko A, Sagara I, Sissoko MS, Guindo O, Diallo AI, Kone M, Toure OB, Sacko M, Doumbo OK., 2008. Impact of intermittent preventive treatment with sulphadoxine-pyrimethamine targeting the transmission season on the incidence of clinical malaria in children in Mali. *Malaria Journal 7*: 123

20. Dobaño C, et al., 2019. A Balanced Proinflammatory and Regulatory Cytokine Signature in Young African Children Is Associated With Lower Risk of Clinical Malaria. *Clinical Infectious Diseases 69*: 820–828
21. Edstein MD, Kocisko DA, Brewer TG, Walsh DS, Eamsila C, Charles BG., 2001. Population pharmacokinetics of the new antimalarial agent tafenoquine in Thai soldiers. *Br J Clin Pharmacol 52*: 663–670
22. Finn TP, et al., 2020. Adherence to Mass Drug Administration with Dihydroartemisinin-Piperaquine and Plasmodium falciparum Clearance in Southern Province, Zambia. *Am J Trop Med Hyg 103*: 37–45
23. Grobusch MP, et al., 2007. Intermittent Preventive Treatment against Malaria in Infants in Gabon—A Randomized, Double-Blind, Placebo-Controlled Trial. *J INFECT DIS 196*: 1595–1602
24. Canavati S ; Guyant P ; Nguon C ; Ly P ; Whittaker M ; Roca-Feltrer A ; Yeung S ;, 2015. Malaria and the mobile and migrant population in Cambodia: A population movement framework to inform strategies for malaria control and elimination. *Tropical Medicine and International Health*
25. Hill J, Hoyt J, Achieng F, Ouma P, L'lanziva A, Kariuki S, Desai M, Webster J., 2016. User and Provider Acceptability of Intermittent Screening and Treatment and Intermittent Preventive Treatment with Dihydroartemisinin-Piperaquine to Prevent Malaria in Pregnancy in Western Kenya. *PLoS ONE 11*: e0150259
26. Houel G., 1954. Prophylaxie médicamenteuse du paludisme par des doses mensuelles de chloroquine et d'amodiaquine. *Bulletin of the Society of Exotic Pathology*
27. Hoyt J, et al., 2018. Intermittent screening and treatment or intermittent preventive treatment compared to current policy of single screening and treatment for the prevention of malaria in pregnancy in Eastern Indonesia: acceptability among health providers and pregnant women. *Malar J 17*: 341
28. Greenwood B., 2008. *IPT in Schoolchildren: Comparison of the Efficacy, Safety, and Tolerability of Antimalarial Regimens in Uganda*. clinicaltrials.gov

29. Huch C, et al., 2018. Assessing and improving the performance of the malaria elimination program at the subnational level in Cambodia. *American Journal of Tropical Medicine and Hygiene*

30. Janssens P G;, 1955. Infantile mortality and malaria in Belgian Congo. [Dutch, English]. *Verhandelingen - Koninklijke Academie voor Geneeskunde van Belgie*

31. Jongdeepaisal M, et al., 2021. Acceptability and feasibility of malaria prophylaxis for forest goers: findings from a qualitative study in Cambodia. *Malaria Journal 20*: 446

32. Jongdeepaisal M, Khonputsa P, Prasert O, Maneenet S, Pongsoipetch K, Jatapai A, Rotejanaprasert C, Sudathip P, Maude RJ, Pell C., 2022. Forest malaria and prospects for anti-malarial chemoprophylaxis among forest goers: findings from a qualitative study in Thailand. *Malaria Journal 21*: 47

33. Jongdeepaisal M, Inthasone S, Khonputsa P, Malaphone V, Pongsoipetch K, Pongvongsa T, Mayxay M, Chindavongsa K, Pell C, Maude RJ., 2022. Forest malaria and prospects for anti-malarial chemoprophylaxis among forest goers: findings from a qualitative study in Lao PDR. *Malaria Journal 21*: 8

34. Kunkel A, et al., 2021. Choosing interventions to eliminate forest malaria: preliminary results of two operational research studies inside Cambodian forests. *Malaria Journal 20*: 51

35. Landier J ; Parker D M; Thu A M; Kajeechiwa L ; Twin M M; Proux S ; Lwin K M; Khin S D; Marta E ; Delmas G ; Nosten F ;, 2016. Relative contribution of generalized early diagnosis and treatment and of targeted mass treatment to elimination of plasmodium falciparum malaria in eastern myanmar. *The American Journal of Tropical Medicine and Hygiene 95*: 1–651

36. Liljander A, Chandramohan D, Kweku M, Olsson D, Montgomery SM, Greenwood B, Färnert A., 2010. Influences of Intermittent Preventive Treatment and Persistent Multiclonal Plasmodium falciparum Infections on Clinical Malaria Risk. *PLOS ONE 5*: e13649

37. Lon C ; Manning J ; Somethy S ; Chann S ; Sriwichai S ; Rekol H ; Dysoley L ; Sinoun M ; Satharath P ; Saunders D ;, 2015. Defining effective, appropriate, implementable strategies for malaria elimination in military forces in Cambodia as a model for mobile populations. *American Journal of Tropical Medicine and Hygiene*

38. Lwin KM, et al., 2012. Randomized, Double-Blind, Placebo-Controlled Trial of Monthly versus Bimonthly Dihydroartemisinin-Piperaquine Chemoprevention in Adults at High Risk of Malaria. *Antimicrob Agents Chemother 56*: 1571–1577

39. Makenga G, et al., 2020. Effectiveness and safety of intermittent preventive treatment for malaria using either dihydroartemisinin-piperaquine or artesunate-amodiaquine in reducing malaria related morbidities and improving cognitive ability in school-aged children in Tanzania: A study protocol for a controlled randomised trial. *Contemporary Clinical Trials Communications 17*: 100546

40. Manning J, et al., 2018. Cluster-randomized trial of monthly malaria prophylaxis versus focused screening and treatment: a study protocol to define malaria elimination strategies in Cambodia. *Trials 19*: 558

41. Manore CA, Teboh-Ewungkem MI, Prosper O, Peace A, Gurski K, Feng Z., 2019. Intermittent Preventive Treatment (IPT): Its Role in Averting Disease-Induced Mortality in Children and in Promoting the Spread of Antimalarial Drug Resistance. *Bull Math Biol 81*: 193–234

42. Maude RJ, et al., 2021. Study protocol: an open-label individually randomised controlled trial to assess the efficacy of artemether-lumefantrine prophylaxis for malaria among forest goers in Cambodia. *BMJ Open 11*: e045900

43. Matangila JR, Doua JY, Mitashi P, da Luz RI, Lutumba P, Van Geertruyden JP., 2017. Efficacy and safety of intermittent preventive treatment in schoolchildren with sulfadoxine/pyrimethamine (SP) and SP plus piperaquine in Democratic Republic of the Congo: a randomised controlled trial. *International Journal of Antimicrobial Agents 49*: 339–347

44. Miller., 1955. Suppression of malaria by monthly drug administration

45. Anon. Mass drug administration of long-acting antimalarials among children in Bossangoa health district, Central African Republic | Mesa. Available at: http://www.mesamalaria.org/mesa-track/mass-drug-administration-long-acting-antimalarials-among-children-bossangoa-health. Accessed

46. Nankabirwa J, Cundill B, Clarke S, Kabatereine N, Rosenthal PJ, Dorsey G, Brooker S, Staedke SG., 2010. Efficacy, Safety, and Tolerability of Three Regimens for Prevention of Malaria: A Randomized, Placebo-Controlled Trial in Ugandan Schoolchildren. *PLoS One 5*: e13438

47. Nankabirwa JI, Wandera B, Amuge P, Kiwanuka N, Dorsey G, Rosenthal PJ, Brooker SJ, Staedke SG, Kamya MR., 2014. Impact of Intermittent Preventive Treatment With Dihydroartemisinin-Piperaquine on Malaria in Ugandan Schoolchildren: A Randomized, Placebo-Controlled Trial. *Clinical Infectious Diseases 58*: 1404–1412

48. Makenga DG., 2019. *Effectiveness and Safety of Intermittent Preventive Treatment for Malaria Using Either Dihydroartemisinin-piperaquine or Artesunate-amodiaquine in Reducing Malaria Related Morbidities and Improving Cognitive Ability in School-aged Children in Tanzania: A Controlled Randomised Trial*. clinicaltrials.gov

49. Nikolov M ; Battle K ; Smith D L; Eckhoff P A; Gething P ; Wenger E A;, 2017. Optimizing highly focal mass drug administration targets for malaria elimination acceleration over networked populations: The case of Haiti. *American Journal of Tropical Medicine and Hygiene 97 (5 Supplement 1)*

50. Rohner F, et al., 2010. In a Randomized Controlled Trial of Iron Fortification, Anthelmintic Treatment, and Intermittent Preventive Treatment of Malaria for Anemia Control in Ivorian Children, only Anthelmintic Treatment Shows Modest Benefit. *The Journal of Nutrition 140*: 635–641

51. Sahan K, et al., 2017. Community engagement and the social context of targeted malaria treatment: a qualitative study in Kayin (Karen) State, Myanmar. *Malar J 16*: 75

52. Silumbe K, et al., 2020. Assessment of the Acceptability of Testing and Treatment during a Mass Drug Administration Trial for Malaria in Zambia Using Mixed Methods. *Am J Trop Med Hyg 103*: 28–36

53. Son DH, et al., 2017. The prevalence, incidence and prevention of Plasmodium falciparum infections in forest rangers in Bu Gia Map National Park, Binh Phuoc province, Vietnam: a pilot study. *Malaria Journal 16*: 444

54. Steketee RW, Wirima JJ, Slutsker WL, Khoromana CO, Breman JG, Heymann DL., 1996. Objectives and methodology in a study of malaria treatment and prevention in pregnancy in rural Malawi: The Mangochi Malaria Research Project. *Am J Trop Med Hyg 55*: 8–16

55. Stingl P., 2004. Malaria: prophylaxis, treatment, stand-by medication. A single mosquito bite suffices

56. Thera MA, Kone AK, Tangara B, Diarra E, Niare S, Dembele A, Sissoko MS, Doumbo OK., 2018. School-aged children based seasonal malaria chemoprevention using artesunate-amodiaquine in Mali. *Parasite Epidemiology and Control 3*: 96–105

57. Tuck JJH, Green AD, Roberts KI., 2005. A malaria outbreak following a British military deployment to Sierra Leone. *J Infect 50*: 171–172

58. Anon. Targeting malaria high-risk populations with tailored intervention packages: A study to assess feasibility and effectiveness in Northern Namibia | Mesa. Available at: http://www.mesamalaria.org/mesa-track/targeting-malaria-high-risk-populations-tailored-intervention-packages-study-assess. Accessed

59. Villegas L ; Hiwat H ; Cairo H ; Hardjopawiro L ;, 2010. Sustained malaria control in Suriname after 3 years of effective interventions. *International Journal of Infectious Diseases*

60. Lorenz von Seidlein, Thomas J. Peto, Rupam Tripura, Christopher Pell, Shunmay Yeung, Jean Marie Kindermans, Arjen Dondorp, Richard Maude. Novel Approaches to Control Malaria in Forested Areas of Southeast Asia: Trends in Parasitology. *Trends in Parasitology 35*: 388–398

61. Wen S, et al., 2016. Targeting populations at higher risk for malaria: a survey of national malaria elimination programmes in the Asia Pacific. *Malaria Journal 15*: 271

62. Xu J-W, Lee R, Li X-H, Liu H., 2021. Transition of radical, preventive and presumptive treatment regimens for malaria in China: a systematic review. *Malar J 20*: 10

63. Staedke SG, Maiteki-Sebuguzi C, Rehman AM, Kigozi SP, Gonahasa S, Okiring J, Lindsay SW, Kamya MR, Chandler CIR, Dorsey G, Drakeley C., 2018. Assessment of community-level effects of intermittent preventive treatment for malaria in schoolchildren in Jinja, Uganda (START-IPT trial): a cluster-randomised trial. *The Lancet Global Health 6*: e668–e679

64. Rehman AM, Maiteki-Sebuguzi C, Gonahasa S, Okiring J, Kigozi SP, Chandler CIR, Drakeley C, Dorsey G, Kamya MR, Staedke SG., 2019. Intermittent preventive treatment of malaria delivered to primary schoolchildren provided effective individual protection in Jinja, Uganda: secondary outcomes of a cluster-randomized trial (START-IPT). *Malar J 18*: 318

65. Clarke SE, Jukes MC, Njagi JK, Khasakhala L, Cundill B, Otido J, Crudder C, Estambale BB, Brooker S., 2008. Effect of intermittent preventive treatment of malaria on health and education in schoolchildren: a cluster-randomised, double-blind, placebo-controlled trial. *The Lancet 372*: 127–138

66. Opoku EC, Olsen A, Browne E, Hodgson A, Awoonor-Williams JK, Yelifari L, Williams J, Magnussen P., 2016. Impact of combined intermittent preventive treatment of malaria and helminths on anaemia, sustained attention, and recall in Northern Ghanaian schoolchildren. *Global Health Action 9*: 32197

67. Marasinghe MM, Karunasena VM, Seneratne AS, Herath HDB, Fernando D, Wickremasinghe R, Mendis KN, Ranaweera D., 2020. Mass radical treatment of a group of foreign workers to mitigate the risk of re-establishment of malaria in Sri Lanka. *Malar J 19*: 346

68. Tseroni M, et al., 2015. Prevention of Malaria Resurgence in Greece through the Association of Mass Drug Administration (MDA) to Immigrants from Malaria-Endemic Regions and Standard Control Measures. *PLoS Negl Trop Dis 9*: e0004215
